# Mechanism of Enzyme Activity Regulation and Strain-Specific Response of *Lentinula edodes* Cultivation Adaptability Under Peach Wood Substrate

**DOI:** 10.3390/jof11090684

**Published:** 2025-09-20

**Authors:** Ning Jiang, Hao-Ran Dong, Long Tian, Tai-Zeng Xin, Shou-Xian Wang, Yu Li, Mei-Na He, Hai-Long Yu

**Affiliations:** 1 Institute of Edible Fungi, Shanghai Academy of Agricultural Sciences, National Engineering Research Center of Edible Fungi, Shanghai 201403, China; jiangning@saas.sh.cn (N.J.); 20180502@saas.sh.cn (H.-R.D.); tl1721923736@163.com (L.T.); xintaizeng0111@163.com (T.-Z.X.); yuli966@126.com (Y.L.); 2 Engineering Research Centre of Chinese Ministry of Education for Edible and Medicinal Fungi, Jilin Agricultural University, Changchun 130118, China; 3 Institute of Plant Protection, Beijing Academy of Agriculture and Forestry Sciences, Beijing 100097, China; wangshouxian@baafs.net.cn

**Keywords:** *Lentinula edodes* cultivation, peach wood substrate, enzyme activity regulation, strain-specific adaptation, arsenic accumulation

## Abstract

The resource utilization of peach wood as agricultural waste holds significant importance for the sustainable development of the edible fungi industry, yet its regulatory effects on the physiology and safety of *Lentinula edodes* (*L. edodes*) remain unclear. This study selected four *L. edodes* (F2, 0912, N5, and 215) and systematically analyzed their cultivation adaptability across five peach wood substrate proportions (0%, 20%, 40%, 60%, and 80%). Results indicated that while high peach wood proportions inhibited laccase activity and delayed mycelial growth, high carboxymethyl cellulase and xylanase activity formed a critical compensatory effect, ultimately enhancing total yield. Peach wood improved production through strain-specific mechanisms. F2 increased via single mushroom weight gain, while N5 relied on xylanase-driven primordia differentiation to boost mushroom numbers. Adding peach wood significantly increased crude protein, crude lipid, and total polysaccharide in F2, maintaining normal agronomic traits and increasing secondary mushroom proportion. Safety risks focused on arsenic accumulation, with 80% peach wood causing F2 to exceed control levels, albeit remaining far below the national standards. This study is among the first to elucidate peach wood’s temporal enzyme regulation for the maintenance of *L. edodes* yield. Future optimization through peach wood pretreatment and low arsenic strain selection could provide technical support for the high value utilization of agricultural waste.

## 1. Introduction

*Lentinula edodes* (*L. edodes*), a classic wood decay fungus, is a large edible and medicinal mushroom. Its fruiting bodies contain significantly lower levels of saturated fatty acids compared to unsaturated fatty acids, with relatively low lipid content, making them a natural low-calorie food [1]. Rich in ergosterol, *L. edodes* effectively prevents rickets in humans [2]. From 1990 to 2020 (over 30 years), mushroom production increased 13.8 times to 42.8 million tons globally [3]. *L. edodes* is the most abundant, followed by oyster mushrooms (*Pleurotus* spp.) [4]. China produces about 93% of the world’s mushrooms, including these species [5]. *L. edodes* remains China’s highest-yield edible variety. According to statistics from the China Edible Fungus Association, the country’s total *L. edodes* production reached 13.0375 million tons in 2023, accounting for 30.08% of the nation’s total edible fungi output. Traditional *L. edodes* cultivation primarily relies on broadleaf tree chips. However, with China’s policy of “improving natural forest protection systems, completely halting commercial logging in natural forests, and increasing forest area and stock volume,” the supply of traditional cultivation substrates has been dramatically impacted. Therefore, developing new cultivation materials to partially replace conventional wood chips holds significant practical importance and value [6].

Sustainable development stands as both a global priority and a critical challenge for humanity in the 21st century [7]. Annual waste production creates significant environmental pollution in agriculture and forestry [8]. Take agricultural byproducts like crop residues and livestock manure as examples: improper disposal not only occupies land resources but also causes ecological imbalance. However, through proper sorting and eco-friendly methods, such as composting and biogas fermentation, these materials can be transformed into valuable resources, reducing environmental impact and enabling sustainable agricultural practices. Rich in organic matter, these waste materials undergo fungal decomposition that enhances nutrient cycling, suppresses pathogen growth, and produces high-quality agricultural byproducts through cultivation techniques. This approach not only boosts waste utilization but also improves ecological conditions, serving as a crucial resource recovery method in green agriculture [9]. Increasingly, researchers are developing innovative substrates for this field, with diverse solutions being actively explored.

Li et al. investigated the effects of culture substrates on the content of flavor components in *L. edodes* and evaluated their sensory quality, providing cultivation insights for obtaining *L. edodes* fruiting bodies with richer taste profiles [10]. Nitta et al. studied the impact of wood chips mixed with poplar on *L. edodes* fruiting body yield, revealing that adding 30% poplar to wood chip culture media could reduce production costs while maintaining fruiting body yields [11]. Kilpatric M’s feasibility trial on spruce bark cultivation revealed sterilization frequency impacts on yield [12]. Leifa’s investigation into coffee husk effects showed that hot water pretreatment increased protein content in *L. edodes* fruiting bodies [13]. Azman et al. produced *L. edodes* using distiller’s grains, finding that mushrooms harvested from distiller’s grains culture had 1.4 times higher average weight and yield compared to those from standard media and conventional substrates, while shortening the cultivation period [14]. Dulce Salmones et al. analyzed multiple cultivation substrates affecting *L. edodes* growth, revealing that two *L. edodes* strains achieved peak yields of 130–133% bioavailability in bagasse, followed by 83–98% bioavailability in sugarcane leaves [15]. V Elisashvili et al. investigated the feasibility of cultivating *L. edodes* on wheat straw and leaves, finding lower yields during leaf biotransformation with 83.4% bioavailability. Cultivating *L. edodes* on wheat straw demonstrated a 95.3% bioavailability rate [16].

Several researchers have investigated the effects of lignin changes on *L. edodes* growth. Wu et al. studied the substrate requirements for *L. edodes* cultivation during its developmental stage based on lignocellulose degradation patterns [17]. Cabrera et al. successfully cultivated *L. edodes* using grape branches and analyzed the relationship between laccase activity and mycelial elongation during cultivation phase [18]. Ding et al. examined the growth of six edible fungi on corn stalks, evaluating lignocellulose degradation. They proposed integrating edible fungi cultivation with corn stalk pretreatment to enhance production potential for various products such as enzymes, sugars, and ethanol [19].

Peach cultivation spans vast areas in China, with China Fruit Circulation Association reporting 12.532 million mu of peach orchards nationwide in 2023. The rapid variety turnover in peach wood necessitates annual pruning to boost yields, resulting in substantial branch waste. Utilizing these branches as biofuel resources has become a critical challenge for developed peach-growing regions. Rich in lignocellulose, peach wood provides ideal substrates for edible fungi cultivation, which helps alleviate the conflict between mushroom cultivation and forest conservation while maintaining ecological balance. As agricultural waste, peach wood costs only 320 yuan per ton, far lower than commercial oak sawdust (650 yuan per ton), which can significantly reduce raw material costs for *L. edodes* cultivation and enhance farmers’ economic benefits. Additionally, the unique physicochemical properties of peach wood provide potential advantages for mushroom cultivation. Fresh branches have a pH value of 5.0–6.0, which aligns with the optimal pH range (5.0–5.5) required for *L. edodes* mycelium growth after crushing and composting, eliminating the need for additional pH regulators. Moreover, the small amounts of active substances like peach glycosides and flavonoids in peach wood can be degraded into low-concentration phenolic compounds through composting and fermentation. This not only prevents the inhibition of *L. edodes* mycelium growth but also suppresses the proliferation of miscellaneous fungi (such as green mold and Alternaria), thereby reducing disease incidence during cultivation.

*L. edodes* strategically differentially expresses genes encoding various extracellular enzymes at distinct growth and developmental stages (e.g., mycelial colonization, browning, primordia formation), thereby exhibiting stage-specific enzyme activity. However, no studies to date have systematically examined strain-specific enzymatic responses of *L. edodes* to peach wood substrates. This study evaluated alternative cultivation methods using peach wood as oak substitutes. Through optimized species selection and formulation development, comprehensive assessments were conducted on production cycles, extracellular enzyme activity, yields, agronomic traits, quality, and safety parameters. This research aims to identify efficient cultivation formulas with peach wood as the primary substrate, to elucidate physiological adaptation mechanisms, and to provide insights for sustainable *L. edodes* industry development alongside resource-efficient utilization of peach wood.

## 2. Materials and Methods

### 2.1. Test Strains and Test Materials

The *L. edodes* strains F2, 215, N5, and 0912 were provided by the National Germplasm Resource Bank (Shanghai, China). The oak sawdust, peach wood chips, poplar wood chips, bran, and gypsum were provided by Shandong Yuyuan Biotechnology Co., Ltd. (Weifang, China). Peach wood was found to be rich in organic matter, containing lignin at a concentration of 130.70 mg/g, cellulose at 602.14 mg/g, and hemicellulose at 223.74 mg/g. The carbon-to-nitrogen (C/N) ratio was determined to be 210.4.

### 2.2. Medium Formulation

Mother stock: PDA medium.

Original seed: 78% wood chips [m (oak wood chips): m (poplar wood chips) = 1:2], 20% wheat bran, and 2% gypsum.

Cultivation types: Control (CK) is the conventional *L. edodes* cultivation formula, and formulas P20, P40, P60, and P80 add different mass fractions of peach wood chips (dry weight) instead of oak wood chips in the conventional formula, respectively. The specific formulas are shown in Table 1.

We mixed all raw materials according to the cultivation formula until thoroughly blended, achieving a moisture content of approximately 57%, using moisture analyzer (Shanghai Difa Instrument & Meter Co., Ltd., Shanghai, China). The mixture was then packed into polypropylene mushroom bags (15 cm × 55 cm × 0.05 cm), with each bag containing about 2100 g of material. Three hundred mushroom bags were produced for each formula. After undergoing 8 h autoclaving at 116 °C pressure, the bags were cooled to room temperature before inoculation. The cultivation bags were incubated at 25 °C in the absence of light. Upon complete colonization of the substrate by mycelium, aeration holes were introduced, and the bags were transferred to light conditions to induce color transformation. Following the completion of color transformation and full maturation of the mycelium, the inner bags were removed to initiate fruiting. Throughout the cultivation process, moisture levels were continuously monitored and maintained through supplemental watering as required. Environmental conditions were maintained within the following ranges: temperature between 15 and 20 °C, relative humidity at 80–90%, and CO_2_ concentration at 500–800 mg/kg [20].

### 2.3. Cultivation Management

The mycelial growth rate was measured using cross-hatching method [21]. Perpendicular lines were drawn through the center of the inoculated block, with their intersection defining the central measurement point. The colony diameter was measured, and the average mycelial growth rate was calculated according to the following formula: Average mycelium growth rate (mm/d) = Colony diameter (mm)/Number of culture days. Three fungal bags were randomly selected and recorded starting from day 10 post-inoculation, with measurements taken every 4 days. Two inoculation holes were chosen for each bag to calculate daily average growth rate of mycelium. The time required for each formula to reach full bag maturity was also statistically analyzed. Due to varying growth periods among strains, bags that had completed color transformation earlier were stored in −3 °C cold storage before being uniformly shelved for *L. edodes* cultivation.

### 2.4. Yield and Agronomic Traits Determination

After mushroom bags were placed on the shelves, each formula bag was divided into 3 groups of 100 bags. Harvesting was conducted when the *L. edodes* fruiting body membrane was about to rupture, with a total of 3 batches of fresh fruiting bodies collected. The yield per batch and total yield were recorded, along with the calculation of biological efficiency ((fresh mushroom weight/dry bag weight) × 100%). According to the industry standard GH/T 1013-2015 for *L. edodes*, the proportion of different grades in each formula was determined (Grade 1: cap diameter > 6 cm; Grade 2: cap diameter < 6 cm, but >4 cm; Grade 3: cap diameter < 4 cm) [22].

Using a digital display vernier caliper (Vogor (Shanghai) Technology Co., Ltd., Shanghai, China) and electronic balance (Sinopharm Chemical Reagent Co., Ltd., Beijing, China), we measured the single mushroom weight, pileus weight, pileus diameter, pileus thickness, stipe length, and stipe diameter of fungal specimens from different cultivation formulas. The cap mass proportion (pileus weight/single mushroom weight) was also calculated. For each formula group, 30 randomly selected specimens were analyzed for agronomic traits, with scatter plots of single mushroom weight distribution across different formulations created.

### 2.5. Dynamic Changes in Extracellular Enzyme Activity

The study evaluated *L. edodes* enzyme activities (including laccase, carboxymethyl cellulase (CMCase), and xylanase) in the mycelial growth phase, pigmentation completion phase, primordia formation phase, and fruiting body development phase across different microbial formulations. Three fungal bags containing 20 g of culture medium were collected, mixed with 100 mL sterile water, and incubated in a 25 °C × 150 r/min shaking bed for 24 h. The fermentation broth was then centrifuged, and the supernatant collected as crude enzyme solution. Laccase determination followed the method by Vetchinkina [23], while CMCase and xylanase assays were conducted according to NY/T 912-2004 [24] and GB/T 23874-2009 [25]. Enzyme activity was detected using a microplate reader (Tecan Trading (Shanghai) Co., Ltd., Shanghai, China). All enzyme activities in this study were expressed in U/mL. All enzyme activity data reported in the manuscript were presented as the mean ± standard deviation (SD) of three biological replicates. The enzyme activity values were normalized to the dry weight of the substrate.

### 2.6. Nutritional Composition of Fruiting Bodies

Determination of nutrient components in F2 fruiting bodies: The second-generation fruiting bodies were collected and dried at 60 °C until a constant weight was achieved. The dried fruiting bodies were crushed and stored at 4 °C for later use. Total polysaccharides were extracted using water extraction and the alcohol precipitation method, with total polysaccharide content determined by the phenol–sulfuric acid method [26]. Ash content and crude lipid content were measured according to GB/T 5009.4-2016 and GB 5009.6-2016 [27,28]. Crude fiber content was determined in accordance with GB 5009.10-2003 [29]. Crude protein content was analyzed using a Kjeldahl nitrogen analyzer (Beijing Yishan Huitong Technology Co., Ltd., Beijing, China) [30]. The data are presented as the mean ± standard deviation (SD) of three biological replicates.

### 2.7. Pesticide Residue and Heavy Metal of Fruiting Bodies

Determination of pesticide residues and heavy metal content in the first fruiting body of F2: The first fruiting body was collected and dried at 60 °C until constant weight. The dried fruiting body was crushed and stored at 4 °C for later use.

We weighed 2 g of dry samples and transferred them into a 50 mL centrifuge tube. After adding 20 mL of acetonitrile, 1 g of sodium chloride, and 4 g of anhydrous magnesium sulfate, the mixture was shaken for 30 min. The suspension was then subjected to 5 min centrifugation at 8000 r/min. The resulting supernatant served as the extraction solution. A 10 mL portion of this solution was dried by nitrogen gas until nearly dry then reconstituted using 8 mL of a 95% dichloromethane–methanol mixture to enhance the storage stability of alkali-sensitive pesticides. Following the purification methods for amino acid mixed adsorbents and GCB, the pre-mixed adsorption mixture was added to the centrifuge tube to purify the extract. The pesticide residues in the fruiting bodies were subsequently analyzed using a liquid chromatography–tandem mass spectrometry (LC-MS) system (Thermo Fisher Scientific, Waltham, MA, USA) [31]. The data are presented as the mean ± standard deviation (SD) of three biological replicates.

We weighed 0.4 g of the sample (accurate to 0.0001 g) and placed it in a polytetrafluoroethylene digestion vessel. After adding 5 mL of nitric acid, the sample was soaked overnight. The inner lid was sealed, and the stainless-steel outer sleeve was tightened. The vessel was then placed in a constant temperature drying oven: 80 °C for 1–2 h, 120 °C for 1–2 h, followed by 160 °C for 4 h. After natural cooling to room temperature, the sample was opened and heated until nearly dry. The digestion solution was transferred into a 25 mL volumetric flask. The inner vessel and lid were washed three times with a small amount of 1% nitric acid solution. The combined wash liquid was transferred to the flask and made up to the mark with 1% nitric acid. The mixture was homogenized and prepared for analysis, while a blank reagent test was conducted simultaneously. The sample solution was then analyzed for arsenic, cadmium, lead, and mercury content using inductively coupled plasma mass spectrometry (ICP-MS) system (Thermo Fisher Scientific, Waltham, MA, USA) [32]. The data are presented as the mean ± standard deviation (SD) of three biological replicates.

### 2.8. Statistical Analysis

Data are expressed as mean ± SD. Data analysis was performed followed by Duncan’s multiple range test (*p* < 0.05) and Pearson correlation, conducted with SPSS 23.0. Figures were generated using GraphPad Prism 9 software.

## 3. Results

### 3.1. Appropriate Proportion of Peach Wood Promotes Mycelial Growth

**Figure 1:**
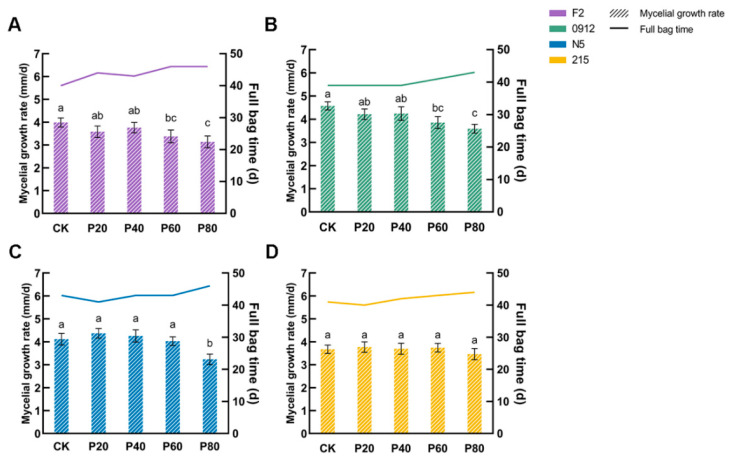
Mycelial growth rate and full bag time of each strain under different formulations. Mycelial growth rate and full bag time of F2 (**A**), 0912 (**B**), N5 (**C**), and 215 (**D**) under different formulations. Values with no letters in common are significantly different (*p* < 0.05).

When peach wood was added at 20% and 40% concentrations, it showed no significant effect on mycelial growth rates of four strains. However, when peach wood concentration reached 60% or higher, all three strains except 215 experienced marked inhibition of mycelial growth (Figure 1). At 60% concentration, the mycelial growth rate of F2 decreased from 3.99 mm/d to 3.38 mm/d, while 0912’s growth rate dropped by 19.54%. When peach wood concentration increased to 80%, F2’s growth rate further decreased to 3.14 mm/d, and 0912 and N5’s rates also declined from their original levels of 4.58 mm/d and 3.67 mm/d to 3.59 mm/d and 3.47 mm/d, respectively. The growth rate of 215 remained unaffected by peach wood concentration. The mycelial growth rate showed a negative correlation with full bag time. Slower growth meant longer full bag time and higher production costs. When 80% peach wood was added, full bag times for F2 and N5 increased from 40 d and 43 d to 46 d and 46 d, respectively. Based on these results, we concluded that high peach wood concentration dramatically inhibited mycelial growth, but this suppression could be mitigated through strain screening.

### 3.2. Enzyme Activity Reveals the Adaptation Mechanism of Peach Wood

**Figure 2:**
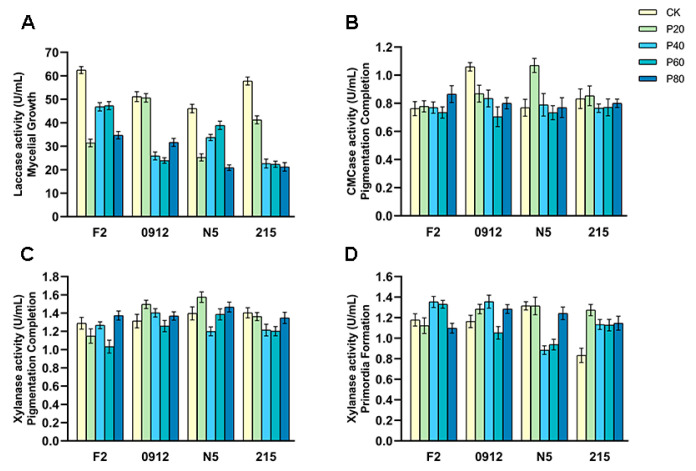
Three key enzyme activities in critical growth periods. Three key enzyme activities of F2, 0912, N5, and 215 in critical growth periods, including Laccase activity in the mycelial growth phase (**A**), CMCase activity in the pigmentation completion phase (**B**), and Xylanase activity in the pigmentation completion phase (**C**) and the primordia formation phase (**D**).

During mushroom growth, fungal mycelium degrades substrate nutrients through extracellular enzyme secretion to support normal development. To further investigate the physiological mechanism of peach wood’s impact on *L. edodes* growth, we measured three key extracellular enzymes produced during fermentation. Laccase provides a nitrogen source for *L. edodes* growth by breaking down lignin in the substrate and releasing aromatic compounds. During the mycelial growth phase, varying peach wood proportions significantly reduced laccase activity compared to control groups, thereby slowing mycelial growth (Figure 2A). Carboxymethyl cellulase (CMCase) decomposes cellulose into glucose, providing carbon sources for biomass accumulation. At the pigmentation completion phase, different peach wood proportions maintained high CMCase activity, ensuring sufficient carbon supply (Figure 2B). Xylanase degrades hemicellulose into xylo-oligosaccharide, which promotes mycelial extension and fruiting body formation. Throughout both pigmentation completion and primordia formation phases, varying peach wood proportions kept xylanase activity above 1 U/mL. Notably, 0912 and N5 peaked at 1.50 U/mL and 1.58 U/mL, respectively, when using 20% peach wood (Figure 2C,D). Enhanced xylanase activity promoted primordia differentiation, ultimately boosting *L. edodes* yield. Taken together, we concluded that although the addition of peach wood to the substrate inhibited the activity of laccase and delayed the growth of mycelium in *L. edodes*, the high activities of CMCase and xylanase in the pigmentation completion and primordia formation phases ensured the carbon source supply and mushroom induction, subsequently maintaining the yield of *L. edodes*.

### 3.3. Strain-Specific Yield Enhancement Effect

**Figure 3:**
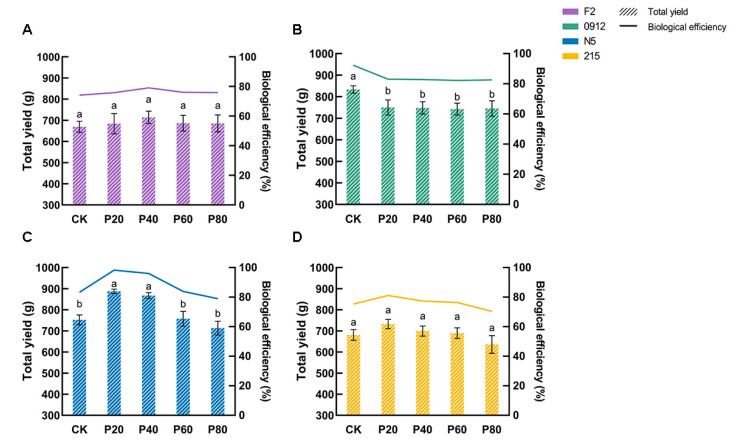
Total yield and biological efficiency of each strain under different formulations. Total yield and biological efficiency of F2 (**A**), 0912 (**B**), N5 (**C**), and 215 (**D**) under different formulations. Values with no letters in common are significantly different (*p* < 0.05).

Enzyme assays revealed compensatory effect of peach wood in inhibiting mycelial growth and maintaining subsequent yield in *L. edodes*. To further verify this, we determined the triple-tide mushroom yield and single mushroom weight of four strains under different proportions of peach wood addition and calculated the total yield as well as the biological efficiency. As shown in Figure 3, F2 achieved a peak total yield of 714.11 g at 40% peach wood proportion, representing a 6.7% increase compared to the control group, with biological efficiency of 79.08%. N5 peaked at 887.44 g yield proportion with 20% peach wood addition, showing an 18% improvement over the control group, with biological efficiency of 98.28%. The yield of 215 showed no significant variation across different peach wood proportions, indicating that 215’s yield was not regulated by peach wood. Notably, 0912’s yield decreased with the increasing peach wood proportion in the substrate, suggesting its unsuitability for peach wood cultivation.

**Figure 4:**
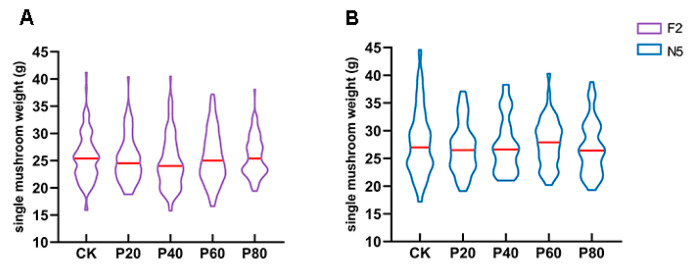
Redistribution map of fruiting body single mushroom weight under different formulations. Redistribution map of fruiting body single mushroom weight of F2 (**A**) and N5 (**B**) under different formulations. Ninety samples were selected from each strain for measurement. The red line represents the median, the top and the bottom of the graph represent the maximum mushroom weight and the minimum mushroom weight, and the width represents the data density.

Analysis of the single mushroom weight in different *L. edodes* strains revealed that F2 exhibited significantly larger single mushroom weights when peach wood was added at 20–40% concentrations, with peak single weight exceeding 40 g. At 20% peach wood concentration, no mushrooms weighing less than 18 g were observed, indicating that F2 primarily boosted yield by increasing individual mushroom weight (Figure 4A). N5 did not produce large mushrooms when peach wood was added, and it demonstrated higher overall single mushroom weight. When peach wood concentration reached 60%, N5 showed a higher median single mushroom weight, suggesting its yield increase stems from enhanced mushroom quantity (Figure 4B). These findings demonstrated that the xylanase-driven compensatory effect manifested through two distinct yield enhancement mechanisms: F2 improved per-mushroom weight; whereas N5 relied on increased mushroom quantity, ultimately leading to total yield differences.

### 3.4. Three-Dimensional Effects of Peach Wood on Quality Enhancement

To further evaluate the impact of peach wood on *L. edodes* quality characteristics, we measured the agronomic traits, commercial grading, and nutritional components of four strains under different peach wood proportions. As shown in Table 2, high peach wood addition remarkably reduced the stipe length in 0912. When the substrate contained 40% peach wood, F2 exhibited a significantly smaller stipe diameter compared to the control group, while N5 showed a higher pileus thickness and cap mass proportion than the control. Conversely, 215 demonstrated reduced stipe diameter but higher cap mass proportion than control. These results indicated that adding peach wood to the substrate had no significant effect on the overall morphology of *L. edodes* fruiting bodies, allowing all strains to grow normally. Meanwhile, low peach wood proportions increased the edible portions of *L. edodes*, improved raw material utilization efficiency, and better met market demands.

**Figure 5:**
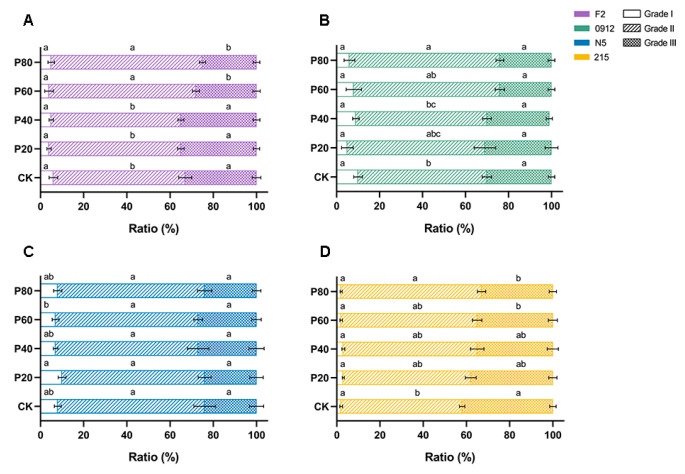
Proportion of mushrooms of various grades of each strain under different formulations. Proportion of mushrooms of various grades of F2 (**A**), 0912 (**B**), N5 (**C**), and 215 (**D**) under different formulations. Values with no letters in common are significantly different (*p* < 0.05).

Grading is essential during the sales process of *L. edodes*. To verify whether peach wood addition affected different grades of *L. edodes* fruiting bodies, we analyzed the proportion of each grade across four strains under varying peach wood supplementation levels. As shown in Figure 5, all four strains maintained consistent proportions of first-grade mushrooms regardless of added peach wood volume. When substrate contained over 60% peach wood, the second-grade mushroom proportion in F2 significantly increased compared to the control group, while the third-grade proportion markedly decreased (Figure 5A). High peach wood supplementation notably boosted second-grade mushroom yield in 215, while reducing third-grade proportion (Figure 5D). The 0912 sample also demonstrated significant growth in second-grade mushroom proportion (Figure 5B). N5 showed no detectable effect from peach wood supplementation on any grade proportions (Figure 5C). The stability in the proportion of first-grade mushrooms across treatments notwithstanding, the marked increase in second-grade mushrooms, occurring alongside a concomitant reduction in third-grade mushrooms, conferred a considerable economic advantage. Within prevailing market structures, second-grade mushrooms commanded a significantly higher unit price than third-grade specimens. Consequently, this alteration in grade distribution indicated an elevation in total output value under consistent yield conditions, thereby enhancing the overall profitability of the cultivation practice. These results collectively indicated that peach wood addition effectively enhanced second-grade mushroom yield, thereby improving market profitability.

*L. edodes*, a high-value food source, is rich in various nutrients. We measured total polysaccharides, crude protein, ash content, crude lipid, and crude fiber levels in F2 under different peach wood addition proportions. As shown in Table 3, when the substrate contained 40% peach wood, crude lipid and crude fiber reached peak values, increasing by 32.4% and 155.0%, respectively, compared to control group. At 60% peach wood proportion, crude protein and ash content peaked, showing increases of 10.1% and 9.5% over the control. Beyond 60% peach wood proportion, total polysaccharide content in F2 increased with higher peach wood proportions. Remarkably, the 80% peach wood addition level boosted total polysaccharide content by 31.4% compared to control. These findings demonstrated that peach wood supplementation enriched nutritional profiles in *L. edodes*, allowing for tailored adjustments in addition proportions to meet specific market demands.

### 3.5. Security Verification

With the improvement of living standards and growing emphasis on product safety, the safety of edible fungi products has increasingly attracted attention. To clarify the impact of peach wood substitution on *L. edodes* safety, we conducted pesticide residue and heavy metal content tests on the first flush of *L. edodes* fruiting bodies and peach wood chips under 80% peach wood addition proportion in F2. We measured seven pesticides commonly used in peach cultivation and ten types specifically emphasized in NY/T 749-2018 “Green Food Edible Fungi”. As shown in Table 4, difenoconazole and imidacloprid were detected in peach wood chips at concentrations of 0.089 mg/kg and 0.038 mg/kg, respectively, while no pesticide residues were found in *L. edodes* fruiting bodies. We speculated that pesticide residues may have been degraded during mycelial growth after high-temperature sterilization.

In heavy metal analysis, we focused on arsenic, cadmium, lead, and mercury levels in *L. edodes* fruiting bodies, with the results shown in Table 5. All treatments demonstrated heavy metal concentrations below the maximum residue limits specified in GB 2763-2021 “Maximum Residue Limits for Pesticides in Foods” and NY/T 749-2018 “Green Food Edible Fungi”. Compared to the control group, peach wood supplementation further reduced heavy metal content in F2. However, when peach wood constituted 80% of the substrate, F2 fruiting bodies showed arsenic levels significantly lower than national standards but higher than control. Similar studies have also been reported in the relevant literature. The contents of the heavy metal mercury, lead, arsenic, and cadmium in the fruiting bodies of *Lepista sordida* grown on rice straw, corncob, and soybean straw substrates varied, but all remained within the national safety range [33]. We speculated that this may be due to arsenic enrichment in peach wood soil, which was efficiently absorbed by mushroom mycelium as water-soluble arsenite. Additionally, the high peach wood proportion altered carbon source composition, promoting arsenic dissolution, and ultimately increasing arsenic content in fruiting bodies. Overall, cultivating *L. edodes* using peach wood as substrate fully complied with food safety requirements.

## 4. Discussion

This study demonstrated that when peach wood content reached ≥60%, the growth rate of mushroom mycelium was significantly inhibited. Specifically, F2 showed a 21.3% reduction in mycelial growth and a 6-day extension in full bag time (Figure 1A). This inhibition directly correlated with decreased laccase activity during the mycelial growth phase (Figure 2A). As a key enzyme in lignin degradation [34], reduced laccase activity restricted nitrogen release and consequently hindered mycelial development. The inhibition of laccase activity by peach wood may originate from its abundance of phenolic compounds (e.g., lignin derivatives or condensed tannins), which can act as natural inhibitors of the enzyme. Notably, during later growth phases (pigmentation completion and primordia formation phases), a dynamic compensatory mechanism involving CMCase and xylanase activities played a crucial role. CMCase continuously degrades cellulose to soluble glucose (Figure 2B), ensuring stable carbon supply and biomass accumulation [35,36]. Xylanase activity peaked at 20% peach wood content (Figure 2C,D), with its hemicellulose-degrading xylo-oligosaccharide serving as an effective mycelial formation inducer [37,38], which explains why strains maintained normal mushroom formation at high peach wood content. Although 215’s laccase activity decreased after peach wood addition, mycelial growth remained unaffected, suggesting that it may possess a unique lignin depolymerization pathway. Future research should prioritize identifying the specific phenolic or terpenoid compounds in peach wood responsible for laccase inhibition and elucidating the stress response pathways activated in the *L. edodes* mycelium. Overall, the “early growth suppression–late development activation” enzymatic temporal regulation model in this study challenges the conventional view that high hardwood content inevitably reduces yields [39]. However, the extended cultivation cycle caused by delayed mycelial growth requires attention. Future efforts should focus on optimizing addition proportions for the targeted breeding of peach wood-tolerant strains.

Peach wood enhanced *L. edodes* yield through strain-specific mechanisms, with the key mechanism being xylanase-mediated fruiting body morphogenesis [16]. F2 achieved a 6.7% yield increase when using 40% peach wood (Figure 3A), characterized by significantly larger individual fruiting bodies (>40 g) and no low-quality mushrooms (<18 g) (Figure 4A). This was attributed to pentosan degradation by xylanase in peach wood, which provided a sufficient carbon skeleton for cap development and ultimately improved cap mass proportion of F2 (Table 2). N5 achieved an 18% yield increase through increased mushroom emergence at 20% peach wood addition, marked by higher median individual mushroom weight while maintaining stable maximum weight (Figure 3C and Figure 4B). This was aligned with the peak xylanase activity (1.58 U/mL) driving primordia differentiation. In contrast, 0912’s yield decreased with increasing peach wood proportion, while 215 showed no significant change (Figure 3B,D), demonstrating the decisive influence of strain genetic background on peach wood response. The dual-path model of “individual mushroom weight gain–increased mushroom count” established based on these results provides theoretical guidance for future precision cultivation.

Peach wood exerted multidimensional effects on *L. edodes* quality. Nutritional analysis showed that substrate supplementation with peach wood increased crude protein content by 10.1%, crude lipid content by 32.4%, and total polysaccharide content by 31.4% in F2 (Table 3). We speculate that the enhancement is attributed to peach wood’s pectin and hemicellulose components, which act as growth promoters. Pectin, a soluble dietary fiber, releases arabinose and galacturonic acid through extracellular enzyme degradation, providing carbon skeletons for protein synthesis [40]. Hemicellulose, when acted upon by xylanase, generates xylo-oligosaccharide, which not only serves as a carbon source for lipid accumulation but also boosts ectopolysaccharide production by activating related genes [41]. Culturally, peach wood addition demonstrated no significant impact on overall fruiting body morphology, supporting normal growth across all strains (Table 2). Notably, F2, 0912, and 215 demonstrated increased secondary mushroom formation (Figure 5), enhancing economic returns. However, safety concerns still persisted. *L. edodes* proved to be a fungus with a high potential for the biodegradation of pesticides [42,43]. Peach wood chips contained 0.089 mg/kg and 0.038 mg/kg of difenoconazole and imidacloprid, respectively, while no residues were detected in *L. edodes* fruiting bodies (Table 4). This may indicate pesticide degradation during high-temperature sterilization. Notably, although the arsenic content in F2 fruiting bodies cultivated with 80% peach wood was significantly higher than in the control, its value (0.311 mg/kg) remained well below the Chinese national standard limit (Table 5) and the Codex Alimentarius Commission (CODEX) guideline. Furthermore, this content level is very low compared to many common foods. For instance, arsenic levels in rice are often several times higher, and daily arsenic intake from drinking water can exceed the intake from these mushrooms. Therefore, based on our data, the health risk to consumers from mushrooms cultivated even with high peach wood proportions is considered negligible. However, the clear accumulation trend indicates that peach wood itself can be a potential source of arsenic. This finding suggests that the pre-use screening of peach wood raw material for heavy metal background levels is necessary. Taking all factors into account, such as food safety, yield, and quality, we recommend a 20–40% substitution ratio as the ideal range for peach wood. This range not only ensures stable yield increase and an improved proportion of commercial-grade mushrooms but also guarantees absolute safety. The use of high ratios (≥80%) should be avoided to prevent extended production cycles and potential arsenic accumulation. There were significant positive correlations between heavy metal concentrations in edible fungi and their substrates, and these relationships can be described with a linear regression model [44]. The existence of these correlations is most likely due to the peach wood’s inherent arsenic accumulation properties and water-soluble arsenite release mechanisms. In the future, risks can be controlled by measures such as peach wood pretreatment (water washing/acid immersion), the avoidance of raw material contamination, and the selection of low enrichment strains.

In summary, this study systematically elucidates the physiological adaptation mechanisms of *L. edodes* cultivated on peach wood. Our findings offer direct guidance for the *L. edodes* industry. Growers can adopt a 20–40% peach wood substitution to partially replace traditional broadleaf wood chips, which can reduce production costs, valorize agricultural waste, and achieve stable or higher economic returns. Future efforts could focus on developing pretreatment processes for peach wood (such as acid–base degradation methods to reduce arsenic/pesticide residues) and breeding specialized strains with laccase tolerance, high xylanase activity, and low arsenic accumulation capacity. The resource utilization of peach wood aligns perfectly with the circular agricultural model of “agricultural waste–edible fungi–organic fertilizer.” The key to successful implementation lies in the tripartite coordinated regulation system of “strain optimization–proportion control–safety assurance,” ultimately achieving a win-win development between the ecological benefits of the edible fungi industry and sustainable resource utilization.

## 5. Conclusions

Our study elucidates the physiological adaptation mechanism of peach wood cultivation in *L. edodes* production. While high peach wood content inhibited laccase activity and delayed mycelial growth, its presence created a critical compensatory effect during the pigmentation completion and primordia formation phases through elevated CMCase and xylanase activities. This dual mechanism ensured a stable carbon supply and fruiting body induction, ultimately boosting total yield. Peach wood enhanced production through strain-specific mechanisms. F2 achieved yield improvement via single mushroom weight gain, while N5 relied on xylanase-driven primordia differentiation to increase mushroom counts. Substrate supplementation with peach wood significantly elevated the crude protein, crude lipid, and total polysaccharide content in F2. Remarkably, high peach wood cultivation maintained normal agronomic traits while increasing secondary mushroom formation. Safety concerns center on arsenic accumulation, with 80% peach wood cultivation producing F2 fruiting bodies containing arsenic levels exceeding those of the control groups. Although well below national standards, attention should nonetheless be paid to the soil enrichment characteristics of peach orchards.

## Figures and Tables

**Table 1:** Cultivation material formula.

Formulation	Oak Wood Chips	Peach Wood Chips	Wheat Bran	Gypsum
CK	80%	0	18%	2%
P20	60%	20%	18%	2%
P40	40%	40%	18%	2%
P60	20%	60%	18%	2%
P80	0	80%	18%	2%

**Table 2:** Agronomic characters of fruiting body of each strain under different formulations. Agronomic characters of fruiting body of F2, 0912, N5, and 215 under different formulations. Data are expressed as mean ± SD. Different lowercase letters in the same column indicate significant difference (*p* < 0.05).

Strain	Formulation	Pileus Diameter (cm)	Pileus Thickness (cm)	Stipe Length (cm)	Stipe Diameter (cm)	Cap Mass Proportion (cm)
F2	CK	5.78 ± 0.02 ^a^	1.31 ± 0.01 ^a^	3.38 ± 0.05 ^b^	1.51 ± 0.06 ^a^	0.7889 ^a^
	P20	5.77 ± 0.04 ^a^	1.29 ± 0.01 ^a^	3.39 ± 0.06 ^b^	1.51 ± 0.03 ^a^	0.7845 ^a^
	P40	5.69 ± 0.04 ^a^	1.27 ± 0.02 ^a^	3.39 ± 0.04 ^b^	1.42 ± 0.03 ^b^	0.7934 ^a^
	P60	5.72 ± 0.09 ^a^	1.29 ± 0.02 ^a^	3.56 ± 0.04 ^a^	1.45 ± 0.06 ^ab^	0.7949 ^a^
	P80	5.76 ± 0.05 ^a^	1.30 ± 0.02 ^a^	3.51 ± 0.03 ^a^	1.44 ± 0.05 ^ab^	0.7935 ^a^
0912	CK	5.91 ± 0.09 ^a^	1.33 ± 0.02 ^a^	3.55 ± 0.04 ^b^	1.55 ± 0.09 ^a^	0.7884 ^a^
	P20	5.94 ± 0.02 ^a^	1.34 ± 0.02 ^a^	3.67 ± 0.05 ^a^	1.61 ± 0.07 ^a^	0.7703 ^b^
	P40	5.88 ± 0.09 ^ab^	1.35 ± 0.04 ^a^	3.39 ± 0.06 ^cd^	1.56 ± 0.12 ^a^	0.7896 ^a^
	P60	5.75 ± 0.09 ^b^	1.31 ± 0.02 ^ab^	3.46 ± 0.02 ^c^	1.47 ± 0.02 ^a^	0.7949 ^a^
	P80	5.76 ± 0.08 ^b^	1.28 ± 0.02 ^b^	3.33 ± 0.05 ^d^	1.52 ± 0.02 ^a^	0.7884 ^a^
N5	CK	5.87 ± 0.09 ^a^	1.39 ± 0.03 ^b^	3.48 ± 0.11 ^a^	1.49 ± 0.04 ^a^	0.8023 ^b^
	P20	5.82 ± 0.14 ^a^	1.42 ± 0.04 ^ab^	3.46 ± 0.02 ^a^	1.42 ± 0.07 ^a^	0.8089 ^b^
	P40	5.85 ± 0.08 ^a^	1.44 ± 0.01 ^a^	3.37 ± 0.05 ^a^	1.43 ± 0.03 ^a^	0.8155 ^a^
	P60	5.91 ± 0.08 ^a^	1.39 ± 0.02 ^ab^	3.37 ± 0.04 ^a^	1.43 ± 0.02 ^a^	0.8138 ^a^
	P80	5.83 ± 0.08 ^a^	1.32 ± 0.02 ^c^	3.23 ± 0.04 ^b^	1.49 ± 0.08 ^a^	0.8125 ^a^
215	CK	5.54 ± 0.09 ^a^	1.34 ± 0.03 ^a^	4.04 ± 0.03 ^b^	1.44 ± 0.02 ^bc^	0.7153 ^c^
	P20	5.57 ± 0.05 ^a^	1.33 ± 0.02 ^a^	4.34 ± 0.09 ^a^	1.43 ± 0.04 ^c^	0.7305 ^b^
	P40	5.55 ± 0.12 ^a^	1.30 ± 0.02 ^ab^	4.11 ± 0.06 ^b^	1.41 ± 0.03 ^c^	0.7452 ^ab^
	P60	5.60 ± 0.12 ^a^	1.27 ± 0.03 ^bc^	4.44 ± 0.15 ^a^	1.50 ± 0.03 ^a^	0.7497 ^a^
	P80	5.44 ± 0.10 ^a^	1.25 ± 0.03 ^c^	3.83 ± 0.03 ^c^	1.49 ± 0.03 ^ab^	0.7420 ^ab^

**Table 3:** Nutrient composition of fruiting body of F2 under different formulations. Nutrient composition of fruiting body of F2 under different formulations. Data are expressed as mean ± SD. Different lowercase letters in the same column indicate significant difference (*p* < 0.05).

Formulation	Total Polysaccharides (mg/g)	Crude Protein (g/kg)	Ash Content (g/kg)	Crude Lipid (g/kg)	Crude Fiber (g/kg)
CK	25.27 ± 1.70 ^c^	250.44 ± 2.86 ^c^	66.53 ± 0.87 ^bc^	64.20 ± 2.45 ^d^	12.03 ± 0.82 ^c^
P20	23.65 ± 1.73 ^c^	261.99 ± 1.48 ^b^	67.23 ± 0.17 ^b^	71.84 ± 0.51 ^c^	19.58 ± 0.61 ^b^
P40	24.01 ± 0.45 ^c^	264.86 ± 0.79 ^b^	71.93 ± 0.49 ^a^	85.03 ± 0.93 ^a^	30.68 ± 4.47 ^a^
P60	29.42 ± 2.50 ^b^	275.81 ± 2.35 ^a^	72.88 ± 0.58 ^a^	78.90 ± 0.50 ^b^	19.29 ± 0.47 ^b^
P80	33.20 ± 1.23 ^a^	241.38 ± 3.89 ^d^	65.76 ± 1.01 ^c^	63.90 ± 1.26 ^d^	14.39 ± 0.96 ^c^

**Table 4:** Pesticide residues in peach wood chips and F2 fruiting bodies.

Analyte	Peach Wood Chips (mg/kg)	Fruiting Bodies (mg/kg)
Difenoconazole	0.089	ND (<0.01)
Abamectin	ND (<0.05)	ND (<0.05)
Acetamiprid	ND (<0.01)	ND (<0.01)
Pyraclostrobin	ND (<0.01)	ND (<0.01)
Methyl parathion	ND (<0.01)	ND (<0.01)
Pyrimethanil	ND (<0.01)	ND (<0.01)
Imidacloprid	0.038	ND (<0.01)
Malathion	-	ND (<0.01)
Dimethoate	-	ND (<0.01)
Cyhalothrin (including λ-cyhalothrin)	-	ND (<0.01)
Cyfluthrin (including β-cyfluthrin)	-	ND (<0.01)
Deltamethrin	-	ND (<0.01)
Cypermethrin (including θ-cypermethrin)	-	ND (<0.01)
Flucythrinate	-	ND (<0.01)
Prochloraz (including prochloraz-manganese)	-	ND (<0.01)
Carbendazim	-	ND (<0.01)
Chlorothalonil	-	ND (<0.01)

**Table 5:** Contents of heavy metals in fruiting body of F2 under different formulations. Contents of heavy metals in fruiting body of F2 under different formulations. Data are expressed as mean ± SD. Different lowercase letters in the same column indicate significant difference (*p* < 0.05).

Formulation	Arsenic (μg/kg)	Cadmium (μg/kg)	Lead (μg/kg)	Mercury (μg/kg)
CK	186.17 ± 4.53 ^b^	1295.42 ± 37.45 ^a^	343.20 ± 9.84 ^b^	6.27 ± 0.27 ^b^
P20	125.76 ± 3.53 ^d^	973.12 ± 49.14 ^d^	388.19 ± 16.53 ^a^	5.55 ± 0.19 ^c^
P40	143.58 ± 4.77 ^c^	1067.41 ± 43.12 ^bc^	301.11 ± 8.89 ^c^	5.47 ± 0.02 ^c^
P60	141.29 ± 4.09 ^c^	1090.15 ± 24.69 ^b^	261.19 ± 8.67 ^d^	6.26 ± 0.28 ^b^
P80	311.13 ± 12.39 ^a^	1021.82 ± 8.35 ^cd^	221.12 ± 5.82 ^e^	7.00 ± 0.34 ^a^
Limited standard	≤1000	≤2000	≤2000	≤200

## Data Availability

Datasets used or analyzed during the current study are available in the manuscript.
